# Analysis of molecular networks and targets mining of Chinese herbal medicines on anti-aging

**DOI:** 10.1186/s12906-016-1513-2

**Published:** 2016-12-28

**Authors:** Qi-yu Jiang, Mei-si Zheng, Xiao-jing Yang, Xiao-sheng Sun

**Affiliations:** Guangzhou University of Chinese Medicine, Airport Road NO.12, Guangzhou, People’s Republic of China

**Keywords:** Anti-aging, Kidney-tonifying, Bioinformatics, Data mining, Molecular Network

## Abstract

**Background:**

Many kidney-tonifying Chinese herbal medicines exert effects on anti-aging by comprehensive interactions of multiple targets. However, the interactions of multi-targets targeted by effective ingredients of kidney-tonifying Chinese herbal medicines are unknown. In this study, to explore the systems pharmacology mechanisms of kidney-tonifying Chinese medicines on anti-aging, we establish the molecular networks with the interactions of multi-targets, analyze bio-functions and pathways with IPA, and calculated the mutual interaction pairs of targets (target pairs) with data mining, respectively.

**Methods:**

Kidney-tonifying Chinese medicines with anti-aging effects were screened from the Chinese Pharmacopoeia and the literatures. Target proteins of these herbal medicines were obtained from bioinformatics databases. Comparisons of molecular networks, bio-functions and pathways given by Ingenuity Pathway Analysis system showed the similarities and the differences between kidney Yin-tonifying herbal medicines and kidney Yang-tonifying herbal medicines. Target pairs with high correlation related to anti-aging were also discovered by data mining algorithm. And regulatory networks of targets were built based on the target pairs.

**Results:**

Twenty-eight kidney-tonifying herbal medicines with anti-aging effects and 717 related target proteins were collected. The main bio-functions that all targets enriched in were “Cell Death and Survival”, “Free Radical Scavenging” and “Cellular Movement”, etc. The results of comparison analysis showed that kidney Yin-tonifying herbal medicines focused more on “Cancer related signaling”, “Apoptosis related signaling” and “Cardiovascular related signaling”. And kidney Yang-tonifying herbal medicines focused more on “Cellular stress and injury related signaling” and “Cellular growth, proliferation and development related signaling”. Moreover, the results of regulatory network showed that the anti-aging related target pairs with high correlated degrees of Kidney Yin-tonifying herbal medicines included TNF-PTGS2, TNF-CASP3, PTGS2-CASP3, CASP3-NOS2 and TNF-NOS2, and that of kidney Yang-tonifying herbal medicines included REAL-TNF, REAL-NFKBIA, REAL-JUN, PTGS2-SOD1 and TNF-IL6.

**Conclusions:**

In this study, we achieved some important targets, target pairs and regulatory networks with bioinformatics and data mining, to discuss the systems pharmacology mechanisms of kidney-tonifying herbal medicines acting on anti-aging. Mutual target pairs related to anti-aging found in this study included TNF-PTGS2, TNF-CASP3, PTGS2-CASP3, CASP3-NOS2, TNF-NOS2, REAL-TNF, REAL-NFKBIA, REAL-JUN, PTGS2-SOD1 and TNF-IL6. Target pairs and regulatory networks of targets could reflect more potential interactions between targets and comprehensive effects on anti-aging. Compared with the existing researches, it was found that the kidney-tonifying herbal medicines may exert anti-aging effects in multiple pathways in this study.

**Electronic supplementary material:**

The online version of this article (doi:10.1186/s12906-016-1513-2) contains supplementary material, which is available to authorized users.

## Background

It’s necessary to reveal the interactions of multiple targets targeted by effective ingredients of anti-aging related herbal medicines. It can help us understand the systems pharmacology mechanisms of herbal medicines on anti-aging and develop novel anti-aging drugs. From the point of the traditional Chinese medicine (TCM), kidney yin is the source of the yin jing of human body, which can nourish and moisten the organs, tissues, muscles, etc. And kidney yang is the foundation of the yang qi of human body which has the function of warming and promoting. Aging is mainly due to the weakness of kidney-Qi of human body and many kidney-tonifying Chinese medicines can improve kidney-Qi to extend lifespan [1]. Kidney-tonifying Chinese medicines including kidney Yin-tonifying medicines and kidney Yang-tonifying medicines, exert anti-aging effects by the comprehensive regulations of human body [1]. According to the “Traditional Chinese pharmacy”, the herbal medicines which belong to kidney meridian tropism in “deficiency-tonifying” section are kidney-tonifying medicines; among these kidney-tonifying medicines, herbs in “Yin-tonifying” section are kidney Yin-tonifying medicines, and those in “Yang-tonifying” section are kidney Yang-tonifying medicines. In this study, effective ingredients of anti-aging related kidney-tonifying herbal medicines as well as their targets were obtained from the international bioinformatics databases and molecular chemistry databases. The main pathways, biological functions and interaction networks of targets were analyzed and compared by IPA (Ingenuity Pathway Analysis) [2] system and the data mining of Mutual target pairs was calculated by common gene-mining algorithm [3]. The common gene-mining algorithm we proposed in the previous studies [3] could be used to discover the association among target proteins of herbal medicine. In the previous studies, a few anti-aging herbal medicine (not just kidney-tonifying herbal medicine) were used as data sets to test the algorithm. The results showed that a small amount of aging-associated genes were able to be found. However, in this study, all kidney-tonifying herbal medicines with anti-aging effects were collected and analyzed with IPA system and common gene-mining algorithm. The pathways and associated target pairs related to aging are discovered in this study.

Aging is related with various factors, such as protein and cellular oxidation, mitochondrial DNA damage, etc. [4–6]. Many research literatures have confirmed that some of the active ingredients of kidney-tonifying herbal medicines have anti-aging effects. For example, Some ingredients of *Eucommia ulmoides* and *Psoralen* (bakuchiol, pinoresinol diglucoside, etc.) can influence the proliferation of human skin fibroblasts and up-regulate the expression level of Col III, Col I, TIMP-1 and TIMP-2 mRNA which all have biological characteristics on anti-aging of skin [7]. The effective ingredients of *Polygonum multiflo*rum can reduce the apoptosis of ovarian cells and delay the aging process of D-galactose aging-mice by regulating the expression of apoptosis related genes and proteins such as Bax, Caspase-3 [8]. *Rehmannia and Medl*ar can improve the pathological changes in hippocampal slice of the aging rats, enhance the expression of Bcl-2 gene and inhibit the overexpression of Bax gene. This will be one of the mechanisms of delaying aging [9]. Now many molecular chemistry databases and bioinformatics databases have been built, such as HIT (Herbal Ingredients’ Targets Database) [10], PubChem [11] and CNPD (Chinese Natural Product Datebase) [12]. These databases can provide a lot of information on bioactivity of targets and ingredients of Chinese herbal medicines. Analytical technology of bioinformatics has also been used in pharmacological researches. Besides, the interactions of targets from the known experimental data and the molecular networks can indicate some pharmacological information, and topological relations [13, 14]. Enrichment analysis is another analytical method which is based on the bioinformatics databases. For instance, enrichment analysis which is based on Gene Ontology (GO) database [15] can present us the pathways and biological processes that the target proteins mainly were enriched in. And this analytical method can also reveal the pharmacology mechanism [16]. Based on the molecular networks and enrichment analysis, the data mining of targets can discover the important mutual target pairs with high association degree in all target proteins [3].

## Methods

### Study design

**Fig. 1:**
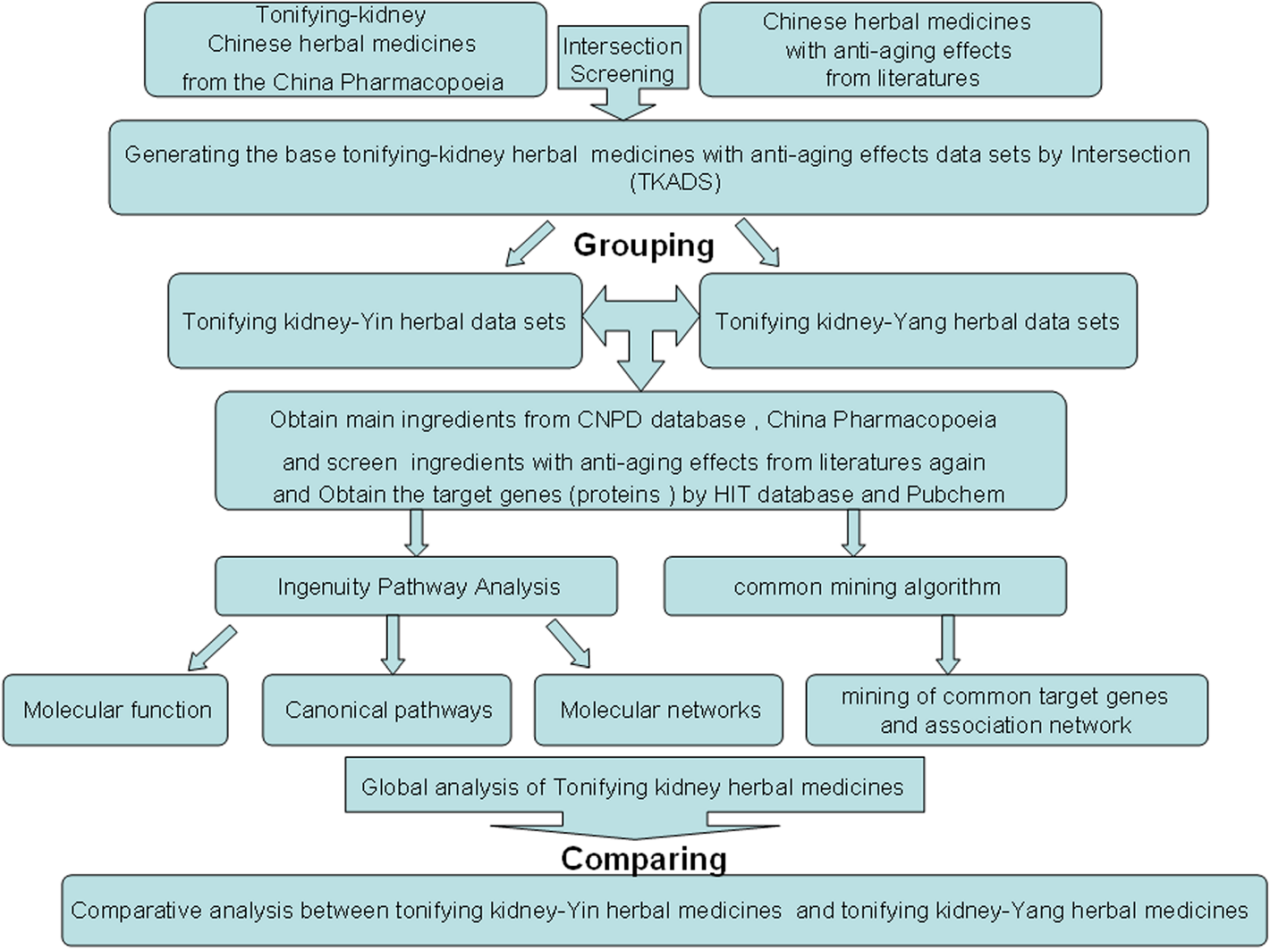
The research framework

The research framework of this study is developed as seen in Fig. 1. It is summarized as follows: (1) Building base data sets of kidney-tonifying herbal medicines with anti-aging effects : According to the Chinese Pharmacopoeia [17], the base herbal data sets were screened and kidney-tonifying Chinese herbal medicines were grouped. Then the obtained herbs were further screened in related research literatures to ensure To ensure that whether they have anti-aging effects. In this way the base kidney-tonifying herbal medicines with anti-aging effects data sets (TKADS) could be built, and they were grouped into kidney Yin-tonifying herbal data sets and kidney Yin-tonifying herbal data sets in accordance with the records of the Chinese Pharmacopoeia. (2) Obtaining the main ingredients and targets: The main ingredients of herbal medicines could be got from CNPD database [12] and the Chinese Pharmacopoeia [17]. As for the target proteins, they could be got from HIT database [10] and Pubchem [11] according to the ingredients. (3) Analysis of molecular networks, pathways and mining of mutual target proteins and regulatory network: Global analysis and comparative analysis between kidney Yin-tonifying herbal data sets and kidney Yin-tonifying herbal data sets were given by IPA system [2] and common mining algorithm [3].

### Data preparation

Kidney-tonifying Chinese herbal medicines were collected as the kidney-tonifying herbal data sets in the “Chinese Pharmacopoeia” [17]. In order to screen out the kidney-tonifying herbal medicines with anti-aging effect, the names for the medicines of herbal data sets and the term “aging” were combined as the search terms in the field of theme, title and keywords in the two literature databases: Pubmed (http://www.ncbi.nlm.nih.gov) [18] and CBM (Chinese biomedical literature database, http://www.sinomed.ac.cn) [19], respectively. Kidney-tonifying herbal medicines with anti-aging effect were confirmed further by reading the research literatures, and then the base kidney-tonifying herbal medicines data sets (TKADS) with anti-aging effect were generated. Two independent reviewers retrived the databases and selected the consistent results. According to the specific attributes of Yin and Yang described in the function section in “the Chinese Pharmacopoeia”, the TKADS were grouped into kidney Yin-tonifying herbal data sets and kidney Yang-tonifying herbal data sets. Similarly all main ingredients of the herbal medicines in TKADS were obtained from CNPD database and the Chinese Pharmacopoeia.

**Fig. 2:**
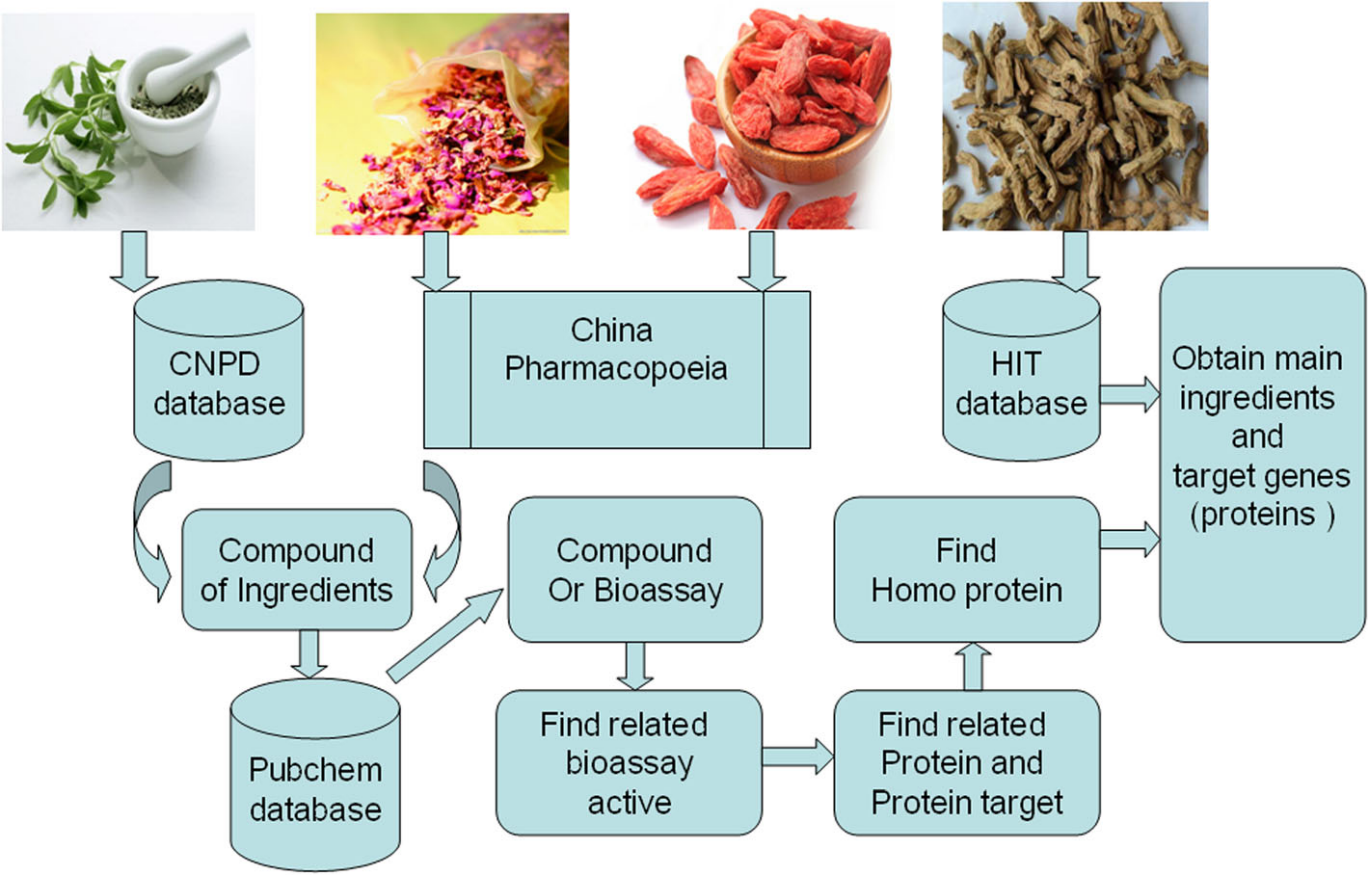
The obtaining targets process

The process of obtaining targets is developed as seen in Fig. 2. HIT [10] database (http://lifecenter.sgst.cn/hit) contains a lot of information on targets and ingredients of many herbal medicines. We got the targets of herbal medicines by retrieving the names and the ingredients of herbal medicines (CAS numbers) in the database. Another way for getting targets is to retrieve PubChem database (http://pubchem.ncbi.nlm.nih.gov). [11], We input the compound names (CAS numbers) of ingredients into the sub-databases “Compound” or “Bioassay” to find “related bioassay active”. If the related targets exist, the table of “related protein and protein targets” would be presented. We finally selected the homo protein targets from all the related protein targets as the final targets of herbal medicines.

### Analysis with IPA

As a biomolecule analysis tool, IPA (Ingenuity Pathway Analysis) [2] system can help to analyze the inputted target data sets. It is based on lots of enrichment and interaction relationships of genes (proteins) which have been confirmed by molecular biologists in the system. In addition, IPA can output some characteristics of inputted gene (protein) data sets with statistical result, such as major biomolecule functions, canonical pathways, molecular interaction networks, etc.

All the target data sets including kidney Yang-tonifying herbal data sets, kidney Yin-tonifying herbal data sets and global herbal data sets were analyzed with IPA. The “core analysis” function in IPA was used to get biomolecule function, canonical pathways and molecular interaction networks of single herbal data sets. The “comparison analysis” function in IPA was used to compare kidney Yin-tonifying and kidney Yang-tonifying herbal data sets. When setting the parameters of all analysis, the species was limited to human, the value of observation curve in tables of canonical pathways was set to “ratio”, and the other parameters were set to default. The top 3 networks with high scores output by IPA were merged together to build the main interaction networks of targets.

### Mining analysis of mutual targets

IPA can help to find the exact pathways and networks that the gene data sets gathered in. However, it is difficult to know which target proteins exactly were mutual and how strong their association degree are in the target data sets. Generally speaking, Kidney-tonifying Chinese herbal medicines exert anti-aging effect by the synthetic action of multi-targets. Each herbal medicine has a certain number of same targets. This phenomenon indicates that there are some mutual and different points in the action mechanisms of these herbal medicines. The kidney-tonifying Chinese herbal medicines had the same anti-aging effect at a macro level, hence it is important to discover the mutual targets related to aging for different herbal medicines. At a certain point, the mutual targets may be the main mechanism of anti-aging for kidney-tonifying Chinese herbal medicines.

**Fig. 3:**
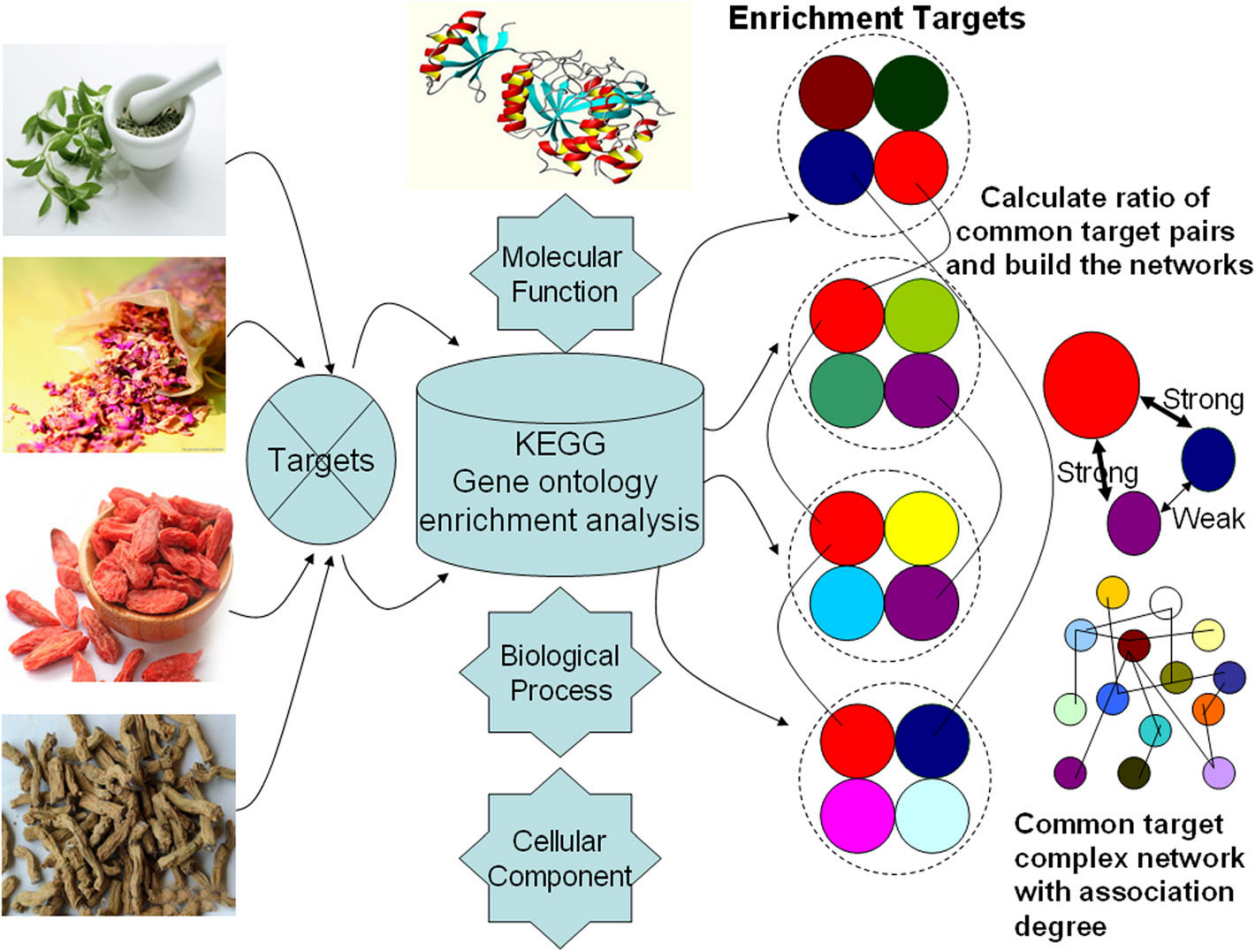
The mining algorithm process

The common gene-mining algorithm has been presented [3] and the algorithm process is developed as seen in Fig. 3. Targets from collected datasets (included kidney Yin-tonifying and kidney Yang-tonifying) were sent to “Bingo” tool [20]. The “Bingo” tool could perform the enrichment analysis to get the enrichment biological environments and the involved targets from the KEGG GO (Gene Ontology) database [15]. “Bingo” is a plugin that widely used to perform functional enrichment analysis as well as clustering the analysis and network comparisons in the field of bioinformatics. Besides it can be connected to the KEGG database. In this study, “Bingo” was mainly used to perform GO enrichment analysis. The enrichment biological environments were molecular function, biological process and cellular component. And *p* value and ratios were used as assessment parameters to describe enrichment biological environments and the aggregation degree of involved targets, respectively. Widely recognized as an evaluation standard in bioinformatics, the *p* value of the enrichment analysis is calculated by Fisher’s test. Importantly, the high enrichment biological environments which most targets mainly enriched in should be focused on. It can be used as a candidate dataset to mine the mutual target pairs among different Chinese herbal medicine because some mutual targets can be found in different biological environments and different herbal medicines. Thus by calculating ratios of all possible combined pairs composed of any two targets, we can get all the target pairs in the high enrichment biological environments. Finally, the mutual targets with high association degree can be discovered and their associative networks can be built.

## Results

According to the method above, 28 kidney-tonifying herbal medicines with anti-aging effect and total 717 related target proteins were collected. There are 335 related target proteins belonging to kidney Yang-tonifying herbal medicines and 382 related target proteins belonging to kidney Yin-tonifying herbal medicines, respectively.(see Additional file 1) The hepatorenal toxicities of all collected herbal medicines were checked in “Toxicologies of Chinese Medicines” [21]. The searching results indicated that most of the collected herbal medicines had no hepatorenal toxicitiy and were safe for human body except for Bu gu zhi (Fructus Psoraleae) and He shou wu (Fallopia multiflora). Bu gu zhi can cause kidney disease if used for a long time [21]. He shou wu can cause liver damage if used for a long time [21]. Some collected sample data of herbal medicines was shown in Table 1. There were some ingredients and many targets in each herbal medicine, Table 1 only showed parts of ingredients and related targets of one herbal medicine.

**Table 1:** Some collected sample data of herbal medicines

Herbal names (Latin)	Hepatorenal toxicity	Effect	Molecular ingredients (CAS number)	Targets
Ba ji tian (*Radix Morindae officinalis*)	None	tonifying kidney-Yang	Aascorbic acid (CAS:1972-08-3)	PTGER3, TGFA, VEGFA, FTMT, SLC11A2, and other 42 targets
Ci wu jia (*Radix seu Caulis et Folium Acanthopana*)	None	tonifying kidney-Yang	Sesamin (CAS:607-80-7)	NOS3, NOX1, UGT1A1, CYP2B6, CCND1, and other 12 targets
Du zhong (*Cortex Eucommiae*)	None	tonifying kidney-Yang	Aucubin (CAS:479-98-1)	BCL2, BAX, TNF, IL6, RELA, NFKBIA, and other 27 targets
Bu gu zhi (*Fructus Psoraleae*)	long-term risks of causing kidney disease	tonifying kidney-Yang	Angelicin (CAS:523-50-2)	CYP1A1, ESR1, ESR2, ESTRB, NR3A2, and other 27 targets
Di huang (*Radix Rehmanniae*)	None	tonifying kidney-Yin	Catalpol (CAS:2415-24-9)	NOS2, SOD1, CASP3, BCL2, ICAM1, and other 14 targets
Nv zhen (*Radix Ligustri lucidi*)	None	tonifying kidney-Yin	Ursolic acid (CAS:77-52-1)	JUN, PTGS2, ACHE, MMP9, RELA, and other 39 targets
Sang shen (*Fructus Mori*)	None	tonifying kidney-Yin	Morin (CAS:480-16-0)	XDH, PIP4K2A, ALOX5, CD36, BATF3, and other 58 targets
Gou qi zi (*Fructus Lycii*)	None	tonifying kidney-Yin	Beta-carotene (CAS:7235-40-7)	CYP3A4, Cyp2b1, CYP1A2, HMOX1, ALB, and other 77 targets

### Results of IPA Analysis

#### Result of global kidney-tonifying herbal medicines with anti-aging effect

The global analysis result with IPA showed that, the top five bio-functions which all targets enriched in were “Cell Death and Survival” (199 targets involved), “Free Radical Scavenging” (104 targets involved), “Gene Expression” (139 targets involved), “Cellular Movement” (152 targets involved) and “Lipid Metabolism” (142 targets involved). The top five related diseases of all target proteins were “Cancer”, “Organismal Injury and Abnormalities”, “Neurological Disease”, “Cardiovascular Disease” and “Metabolic Disease”. Besides, the detailed datas of the top five interaction networks of global targets were shown in Table 2 (see Additional file 2), “score” in Table 2 denotes contribution values of targets involved in.

**Table 2:** The data of top five interaction networks of global targets

Targets in network	Score	Top related Bio Functions and Diseases
ALB, APOB, ATF2, AUH, BCL2, CD40LG, CREB1, CUTA, DDIT3, ELANE, IL6, and other 62 targets	38	Cell Death and Survial, Cardiovascular Disease, Gene Expression
ALOX5, CAV1, CCND1, CDK2, CDK4, CRYZ, CTNNB1, EDN1, EGR1, ESR2, FGF2, and other 47 targets	27	Cancer, Organismal Injury and Abnormalities, Cellular Development
CSD2, DBI, DUOX2, F3, HMOX1, ICAM1, IL4, IL10, IL13, MAOA, MIOX, NOS2, and other 39 targets	26	Free Radical Scavenging, Cell to Cell Signaling and Interaction, Cardiovascular Disease
APP, AR, CASO3, CDKN1A, CXCL8, EGF, FASLG, FN1, HIF1A, IL2, ITGAM, MAPK1, and other 86 targets	25	Gene Expression, Cell Death and Survial, Cancer
ACTA2, ATPC1, BTK, CAPN1, CCL20, CTSB, IL1A, ITGAL, ITGB1, KCNMA1, TP53, and other 106 targets	22	Cellular Growth and Proliferation, Connective Tissue Development

#### Result of kidney Yang-tonifying herbal medicines and kidney Yin-tonifying herbal medicines with anti-aging effect

##### Pathways and networks of kidney Yin-tonifying herbal medicines and kidney Yang-tonifying herbal medicines with anti-aging effects

**Fig. 4:**
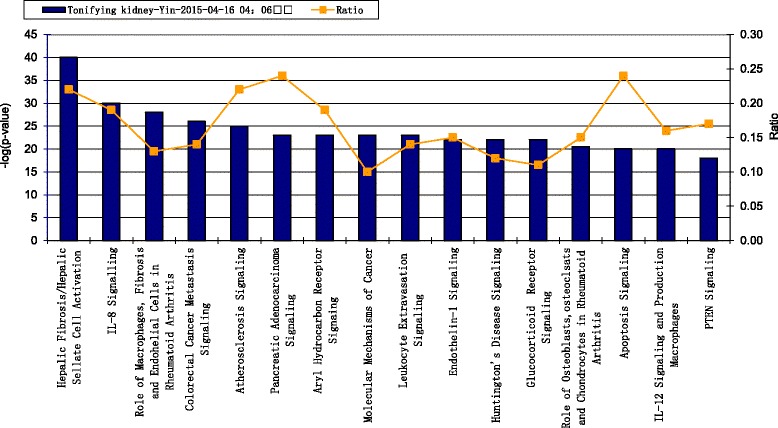
The main canonical pathways of tonifying-kidney-Yin herbal medicines with anti-aging effects. The left Y axis in the figures denoted –log (*p*) values. The right Y axis and curve in the figures, denoted ratios of targets involved in one pathway

**Fig. 5:**
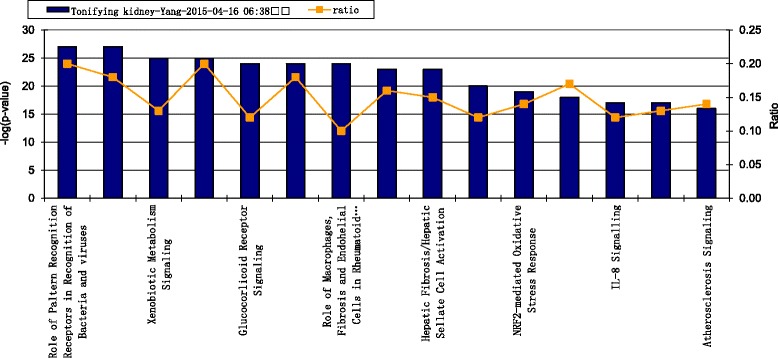
The main canonical pathways of tonifying-kidney-Yang with herbal medicines anti-aging effects. The left Y axis in the figures denoted –log (*p*) values. The right Y axis and curve in the figures, denoted ratios of targets involved in one pathway

The canonical pathways of kidney Yin-tonifying and kidney Yang-tonifying herbal medicines with anti-aging effects were shown in Figs. 4 and 5. The figures only show top 15 according to *p*-value. *P*-value was a significant value given by the statistical method “Fisher exact test”, which showed the concentricity of target proteins involved in one pathway. The higher the –log (*p*) value was, the more significant the relevant pathway would be. In this study the threshold of *p* value was 0.05. In the main pathways of kidney Yin-tonifying herbal medicines with anti-aging effect, the pathways with high ratios included “Apoptosis signaling”, “Atherosclerosis signaling” and “Pancreas adenocarcinoma signaling”. In the main pathways of kidney Yang-tonifying herbal medicines with anti-aging effects, the pathways with high ratios included “Role of pattern-recognition receptors”, “Aryl hydrocarbon receptor signaling” and “HMGB1 signaling”.

**Fig. 6:**
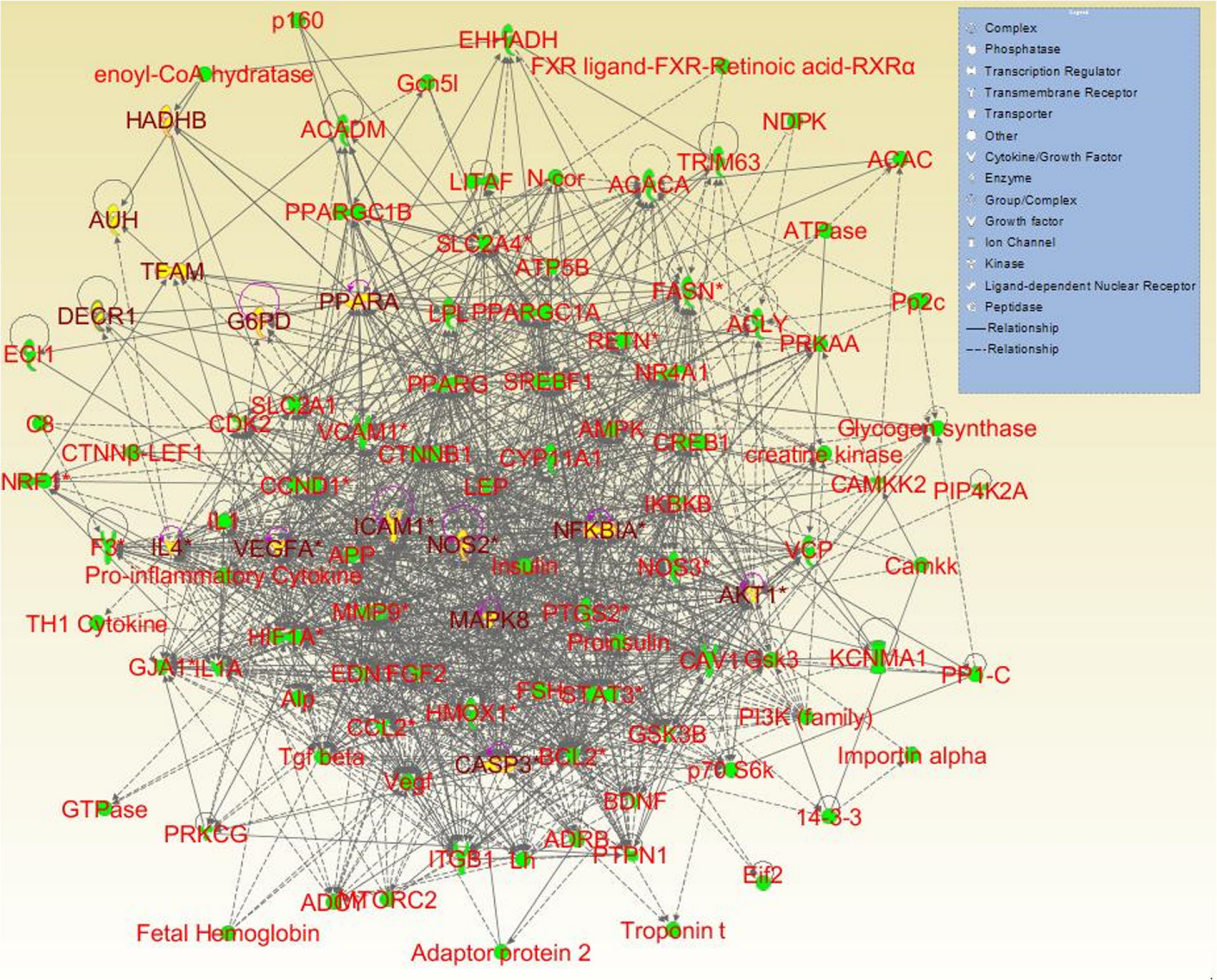
The target network of tonifying-kidney-Yin herbal medicines with anti-aging effects. Nodes denoted targets, and the connections between them denoted interactions between them

**Fig. 7:**
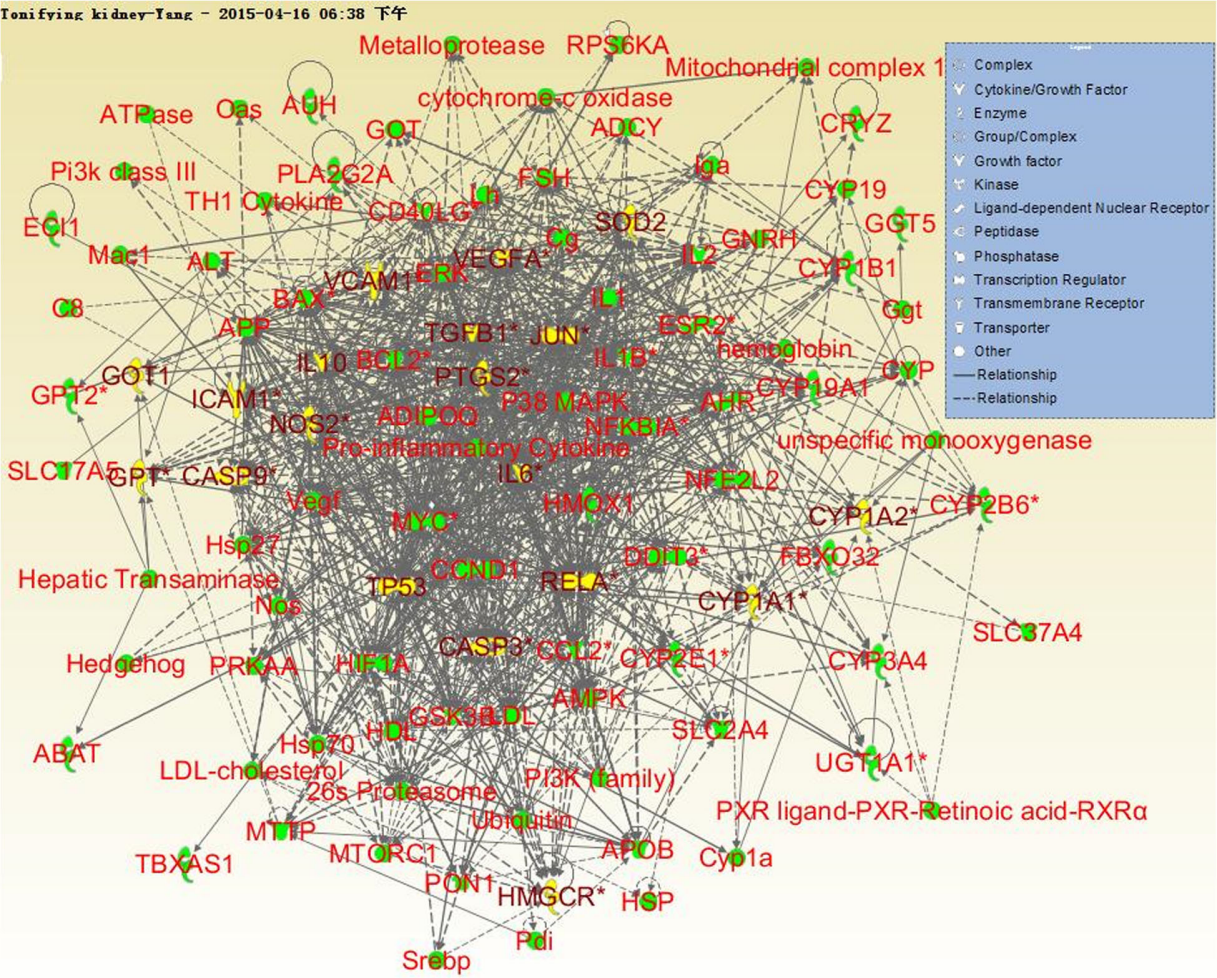
The target network of tonifying-kidney-Yang herbal medicines with anti-aging effects. Nodes denoted targets, and the connections between them denoted interactions between them

The interaction networks of targets of kidney Yin-tonifying and kidney Yang-tonifying herbal medicines with anti-aging effect were shown in Figs. 6 and 7. In these figures, the nodes with yellow and brown color were mutual genes in kidney Yin-tonifying and kidney Yang-tonifying herbal medicines with anti-aging effect. The mutual genes in the target network of kidney Yin-tonifying herbal medicines with anti-aging effect included: CASP3, DECR1, ICAM1, IL4, MAPK8, NFKBIA, PPARA, NOS2, VEGFA, AKT1, AUH, G6PD, HADHB, NRF1 and TFAM. The mutual genes in the target network of kidney Yang-tonifying herbal medicines with anti-aging effect included: CASP3, ICAM1, IL6, JUN, RELA, TGFB1, TP53, VCAM1, CYP1A1, CYP1A2, GGT5, GOT1, GPT, HMGCR, CASP9, NOS2, IL10, PTGS2, SOD2, and VEGFA.

##### Comparison of pathways between kidney Yin-tonifying herbal medicines and kidney Yang-tonifying herbal medicines with anti-aging effect

**Fig. 8:**
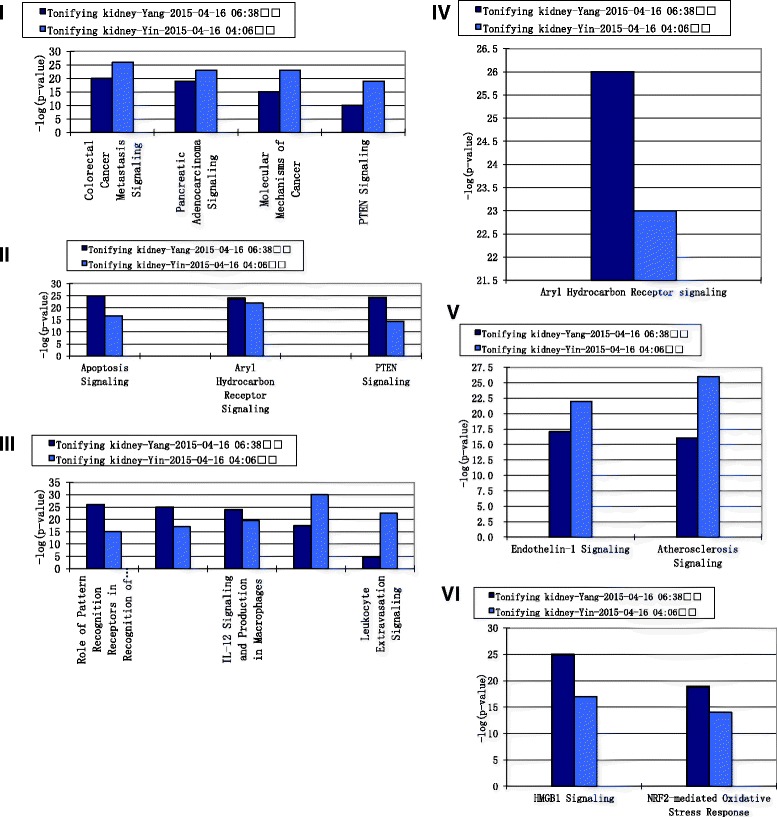
Comparison of pathways between kidney Yin-tonifying herbal medicines and kidney Yang-tonifying. *Blue columns* denoted –log (*p*) values of Yin-tonifying herbal medicines, *brown column* denoted –log (*p*) values of Yang-tonifying herbal medicines. (1) Cancer related signaling. (2) Apoptosis related signaling. (3) Cellular immune response related signaling. (4) Cellular growth and development related signaling. (5) Cardiovascular related signaling. (6) Cellular stress and injury related signaling

According to the top bio-functions with high score and the top pathways with high ratio above, the following specific pathways were chosen for comparison: Apoptosis related signaling, Cancer related signaling, cardiovascular related signaling, cellular growth, proliferation and development related signaling, cellular immune response related signaling, and cellular stress and injury related signaling. The comparisons of pathways were shown in Fig. 8. The significant pathways in apoptosis related signaling included apoptosis signaling, aryl hydrocarbon receptor signaling and pten signaling. Apoptosis signaling and pten signaling in kidney Yin-tonifying herbal medicines were more significant than those in kidney Yang-tonifying herbal medicines. The significant pathways in cancer related signaling included colorectal cancer metastasis signaling, pancreas adenocarcinoma signaling, molecular mechanism of cancer and pten signaling. All the signaling in kidney Yin-tonifying herbal medicines were more significant than those in kidney Yang-tonifying herbal medicines. The significant pathways in cardiovascular related signaling included atherosclerosis signaling and endothelin-1 signaling, which was more significant in kidney Yin-tonifying herbal medicines. In cellular growth, proliferation and development related signaling, aryl hydrocarbon receptor signaling was the only one significant pathway. In the cellular immune response related signaling, the IL-8 signaling and laukocyte extravasation signaling in kidney Yin-tonifying herbal medicines were more significant than those in kidney Yang-tonifying herbal medicines. However, IL-12 signaling, as the role of pattern-recognition receptors and HMGB1 signaling in kidney Yang-tonifying herbal medicines, was more significant than those in kidney Yin-tonifying herbal medicines. In cellular stress and injury related signaling, the significant pathways mainly included HMGB1 signaling and NRF-2 mediated oxidative stress response.

### Result of data mining of mutual target proteins

#### Result of enrichment analysis

Enrichment analysis was performed to get base data items before data mining. Each data item output by “Bingo” included detailed biological that processes targets focused on, *P* values of statistics, ratios of target proteins and specific involved targets [20]. Part of the enrichment data of targets of *Radix Morindae officinalis* was shown in Table 3 (see Additional file 3).

**Table 3:** Part of the enrichment data of target proteins of *Radix Morindae officinalis*

Biological process	*P* value	Ratio	Involved targets
response to chemical stimulus	7.95E-15	0.61	TNF, CCL2, PTGS2, TNC, TH, MME, TGFB1, VCAM1, and other 5 targets
response to stress	8.55E-13	0.61	TNF, CCL2, PTGS2, TNC, TH, MME, TGFB1, SLC11A2, and other 12 targets
response to stimulus	1.66E-11	0.77	TNF, CCL2, PTGS2, TNC, TH, MME, TGFB1, VCAM1, and other 6 targets
response to inorganic substance	1.66E-11	0.29	CUTA, PTGS2, CYP1A1, OLR1, RELA, TNC, SOD1, SOD3, and other 11 targets.
response to wounding	4.25E-10	0.35	IL6, TNF, CCL2, OLR1, CYP1A1, RELA, TNC, MME, and other 9 targets
regulation of immune system process	1.90E-09	0.31	ICAM1, IL6, TNF, CCL2, RELA, SOD1, TGFB1, VCAM1, and other 7 targets
response to drug	1.19E-08	0.24	CCL2, TNF, CYP1A1, PTGS2, JUN, RELA, MME, IL1B, and other 13 targets
positive regulation of response to stimulus	1.77E-08	0.24	IL6, CCL2, TNF, PTGER3, PTGS2, CD40LG, RELA, and other 17 targets

### Result of data mining

**Fig. 9:**
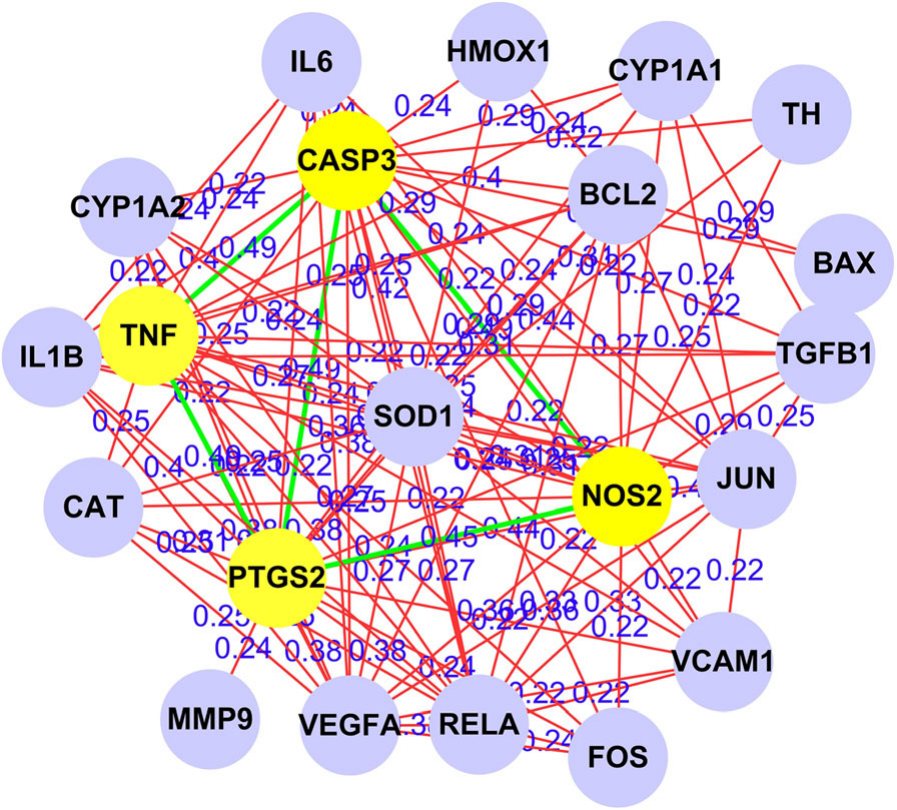
The regulatory network of mutual targets of tonifying-kidney-Yin herbal medicines with anti-aging effects, the nodes denoted common targets, the connection and value between two nodes denoted association degree of a common target pair. The *yellow* nodes and nodes connected with *green lines* were with high association degrees in all common targets

**Fig. 10:**
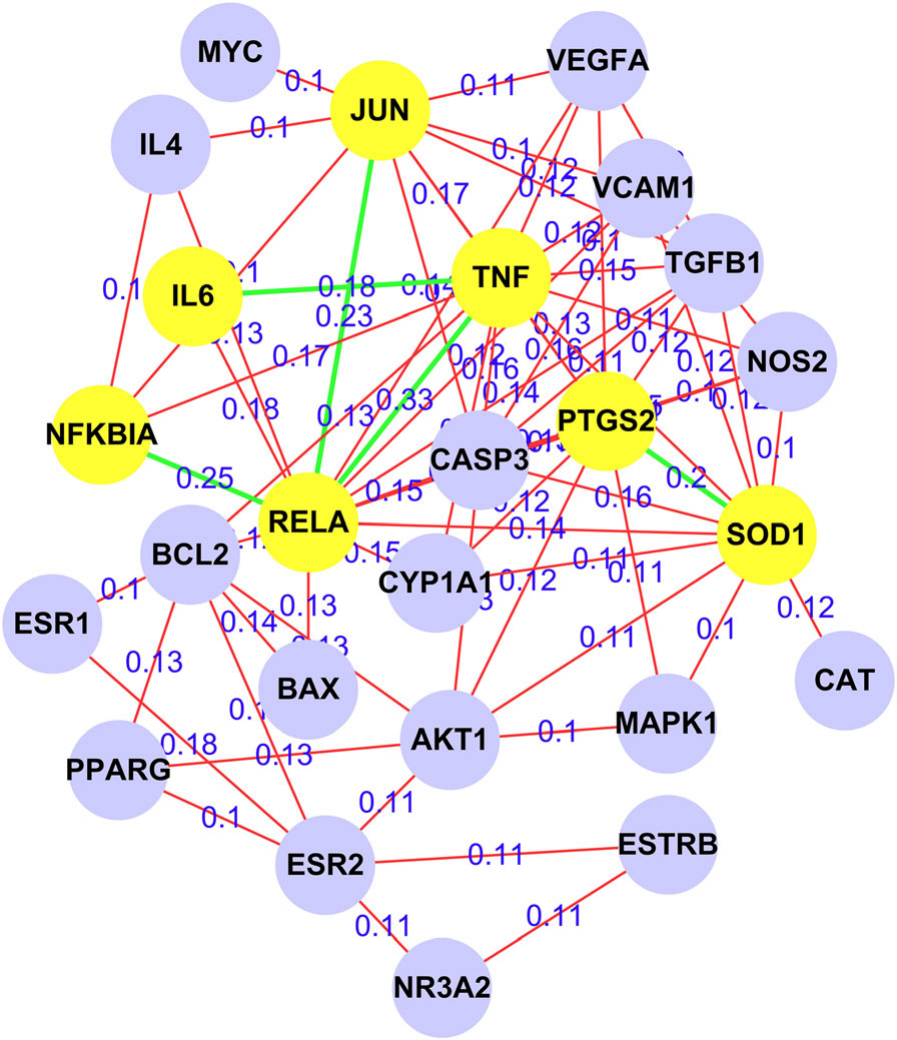
The regulatory network of mutual targets of tonifying-kidney-Yang herbal medicines with anti-aging effects

In this study, the data items with the *P* value in the top 30 and the ratio more than 0.3, were selected for data mining (the maximum *P* value was also less than 1.20E-05). The results (see Additional file 4 and Additional file 5)) were shown in Figs. 9 and 10 by common gene-mining algorithm [3]. The figures only showed pair nodes with association degree more than 0.1. In the figure of kidney Yin-tonifying herbal medicines with anti-aging effect, the mutual target pairs with high association degrees (top 5) included TNF-PTGS2 (0.49), TNF-CASP3 (0.49), PTGS2-CASP3 (0.49), CASP3-NOS2 (0.49) and TNF-NOS2 (0.45). In the figure of kidney Yang-tonifying herbal medicines with anti-aging effect, the mutual target pairs with high association degrees (top 5) included REAL-TNF (0.33), REAL-NFKBIA (0.25), REAL-JUN (0.23), PTGS2-SOD1 (0.2) and TNF-IL6 (0.18). The regulatory networks of anti-aging related targets were built which were based on target pairs, and shown in Figs. 9 and 10.

## Discussion

### Important targets, target pairs and regulatory networks related to anti-aging found in this study

Many existing studies have researched mechanisms of anti-aging of herbal medicines at a target proteins level. However, most of these studies focused on a single or a few targets. Compared with the existing researches, important targets, target pairs and regulatory networks of kidney-tonifying herbal medicines related to anti-aging have been found by data mining in this study. Figures 9 and 10 show the topological relationships and importance of interactions in cells and biological processes. The mining results showed that the important target pairs mainly included TNF-PTGS2, TNF-CASP3, PTGS2-CASP3, CASP3-NOS2, TNF-NOS2, REAL-TNF, REAL-NFKBIA, REAL-JUN, PTGS2-SOD1 and TNF-IL6. These pairs may play an important synergistic role on anti-aging or have close associations with anti-aging. In the target network, nodes with a lot of links and high association degrees to others may be core targets which may play the most key roles on anti-aging. The Fig. 9 shows that in the regulatory networks of targets of kidney Yin-tonifying herbal medicines, the main association centers are TNF, CASP3 and NOS2. And Fig. 10 shows that in the regulatory networks of targets of kidney Yang-tonifying herbal medicines, the main association centers are RELA, SOD1, TNF and JUN. The SOD1 is a known gene related to anti-oxidative stress. Are other genes in results not strong enough to be relevant to aging? In fact, other genes also may strongly relate to aging indirectly. A few studies are beginning to reveal relationships between aging and other genes. TNF is a known gene related to anti-tumor. However, a new study shows that an important enzyme related to anti-aging called SIRT6 will enhance the release of TNF on anti-aging response [22]. Furthermore, another new study shows that NOS could inhibit the expression of a longevity gene called SIRT1. And SIRT1 can inhibit the expression of NFKB and P53 which was encoded by RELA gene. NFKB and P53 may form a regulation network on anti-aging [23]. NOS2 also has been confirmed to be closely associated with skin aging of human body [24]. The expression of C-JUN protein encoded by JUN gene in hippocampus will increase with aging of mice [25]. Although we can not obtain more details on anti-aging of the rest of the target proteins in results, the mining results still show the objective relationships among target proteins. The important targets and their regulatory networks found in this study may point out some directions of the further research for us on anti-aging in the future.

### Multi pathways of kidney-tonifying herbal medicines found in this study may related to aging

At present, a large number of evidence related to anti-aging of kidney-tonifying herbal medicines mainly invlove two fileds: (1) Improving the antioxidant capacity and reducing the production of free radical. (2) Influences on cell apoptosis. For example, there have been an increasing number of studies focusing on the bio-activities of Cistanche species, known as Rou Cong-Rong in Chinese, including antioxidation, neuroprotection and anti-free radicals [26]. As the primary active components of Lycium barbarum berries (Gouqizi), Lycium Barbarum polysaccharides (LBPs) have been reported to have antioxidative and anti-aging properties in different models [27]. Many studies have also confirmed that kidney-tonifying herbal medicines have influences on cell apoptosis. For example, Heshouwu (Fallopia multiflora) has been certified to exert anti-aging effects on the testis by means of inhibiting the occurrence of apoptosis in spermatogenic cells and regulating the expression of key genes in the mitochondrial apoptosis pathway [28, 29]. What’s more, an animal study has revealed that glossy privet fruit (Ligustrum lucidum Ait.), a kidney-tonifying herbal medicine, can inhibit the neural cell apoptosis following the onset of vascular dementia by reducing apoptotic signals induced by cerebral ischemia/hypoxia [30]. Some studys have shown that Cistanche deserticola Ma and Fructus Lycii can inhibit cell apoptosis in the hippocampus and reduce DNA injury to achieve anti-aging [31].

However, this study found that kidney-tonifying herbal medicines may achieve anti-aging in the following multiple pathways:(1) Compared with the existing researches, more antioxidant related pathways and cell apoptosis related pathways have been disclosed in this study. According to the analytical results of Table 2 and Fig. 8 in this study, kidney-tonifying herbal medicines involved the regulation of cellular stress and injury. The detailed pathways mainly included free radical scavenging signaling, HMGB1 signaling and NRF-2 mediated oxidative stress response. And the significant pathways of kidney-tonifying herbal medicines in apoptosis related signaling included apoptosis signaling, aryl hydrocarbon receptor signaling and pten signaling. A few studies have supported part of the points mentioned above. For example, Epimedium can activate antioxidant stress pathways of cells, enhance NRF-2 response, increase GSH levels and reduce ROS released by starting the GCL gene transcription [32]. Another study has shown that an prostate cancer prescription which mainly included kidney-tonifying herbal medicines (Radix Polygoni Multiflori, Radix Ligustri lucidi, Fructus Psoraleae, etc.) may inhibit tumor cells proliferation by promoting PTEN activation to promote apoptosis of tumor cells [33]. This study has also revealed that the relevance between aging and HMGB1 signaling or aryl hydrocarbon receptor signaling may be worth studying in the future.(2) In addition to antioxidant related pathways and cell apoptosis related pathways, other pathways of kidney-tonifying herbal medicines which may have association with aging were also disclosed in this study. These pathways mainly included cancer signaling pathways, cardiovascular signaling pathways and immune response signaling pathways. In Fig. 8, the cancer related signaling pathways of kidney-tonifying herbal medicines included colorectal cancer metastasis signaling, pancreas adenocarcinoma signaling, molecular mechanism of cancer and pten signaling. Cardiovascular disease mainly referred to two signaling pathways of kidney-tonifying herbal medicines including atherosclerosis signaling and endothelin-1 signaling. In the immune response related signaling pathways in Fig. 8, the kidney-tonifying herbal medicines included signaling of HMGB1, signaling of IL-8, signaling of IL-12, etc. Compared with the existing researches of aging, these pathways seemed to be unrelated to aging. However, a few experiments have pointed out some evidence. that atherosclerosis signaling may relate to the aging of arterial and be inhibited by controlling the target ApoB [34]. A kidney-tonifying herbal medicine “Semen Sesami Nigrum” could inhibit ApoB and reduce LDL protein to prevent atherosclerosis [35]. Some other studies have also shown that kidney-tonifying herbal medicines such as Radix Rehmanniae could enhance the level of IL-12 to improve the immunity of aging mice and to inhibit the aging of immune system [36]. So it’s interesting for us to do further exploration to discover the relationships between aging and above pathways.

### Limitations

This study presented the systems pharmacology mechanisms of anti-aging of Kidney-tonifying herbal medicines and discovered the associations among targets of Kidney-tonifying herbal medicines as well as building the regulatory networks of these targets. However, the results were based only on the existing known chemical compositions and targets. In the future, if more unknown ingredients and targets be found, the research results will be richer than now with the analysis methods proposed in this study.

## Conclusions

On the view of the traditional Chinese medicine, many kidney-tonifying herbal medicines can prolong lifespan. Modern researches have confirmed that lots of ingredients of kidney-tonifying herbal medicines have the effects of anti-aging. However, systems pharmacology mechanisms of these herbals have not been presented. In this study, targets of kidney-tonifying herbal medicines were obtained by bioinformatics databases. And analysis of molecular networks and pathways were presented to show the similarities and differences between kidney Yin-tonifying herbal medicines and kidney Yang-tonifying herbal medicines. In addition, some mutual interaction pairs of target proteins (target pairs) were also discovered by data mining. Regulatory networks of targets were built based on these target pairs. In general, Kidney-tonifying herbal medicines may achieve anti-aging in multi-directions including the prevention of aging-related diseases and serious illnesses, the regulation of immune response, the controlling of cell growth and apoptosis, and the regulation of cellular stress and injury. The important mutual pairs of target proteins related to anti-aging may include TNF-PTGS2, TNF-CASP3, PTGS2-CASP3, CASP3-NOS2, TNF-NOS2, REAL-TNF, REAL-NFKBIA, REAL-JUN, PTGS2-SOD1 and TNF-IL6.

## Additional files

Additional file 1: Table S1. The data sets of all collected targets. (XLS 43 kb)
Additional file 2: Table S2. The top five interaction networks involved by targets of Kidney-tonifying Herbal Medicines. (XLS 17 kb)
Additional file 3: Table S3 Part of the enrichment data of targets of *Radix Morindae officinalis*. (XLS 16 kb)
Additional file 4: Table S4 Mining results of kidney Yang-Tonifying Chinese Herbal Medicines. (XLS 222 kb)
Additional file 5: Table S5. Mining results of kidney Yin-Tonifying Chinese Herbal Medicines. (XLS 605 kb)

## Abbreviations

CNPD: Chinese Natural Product Datebase
GO: Gene Ontology
HIT: Herbal Ingredients’ Targets Database
IPA: Ingenuity Pathway Analysis
TCM: Traditional Chinese medicine
